# Experimental study on alkali reduction of film-coated porous ecological concrete by microbial calcium carbonate precipitation technology

**DOI:** 10.1038/s41598-023-50637-9

**Published:** 2024-01-02

**Authors:** Bingxia Wang, Bo Yang, Lu Wang, Bo Lian, Xiaolei Wu

**Affiliations:** Western Centre of Disaster Mitigation in Civil Engineering, Ministry of Education, Lanzhou University of Technology, Lanzhou, 730050 China

**Keywords:** Civil engineering, Renewable energy

## Abstract

Some plants do not grow due to the high pH levels of ecological concrete pore solutions. In this paper, we design and build an integrated device featuring a combined microbial film and a transverse/U-shaped grouting film. We have applied to the China Intellectual Property Office for an invention patent on this device. The device overcomes the blockage of the grouting port caused by microbial and vertical grouting. The vertical grouting tube leaves holes inside a specimen, reducing the compressive strength, while the integrated device optimizes and decreases variation in the recycling of microbial bacteria. The reduction in the pore alkalinity of porous ecological concrete resulting from the microbial grouting film of this device is larger than that resulting from a microbial sprayed film. The pH values of porous ecological concrete with microbial grouting films and microbial sprayed films are obtained by the pure slurry soaking method and solid–liquid extraction method, respectively. The pH value is lower for the film obtained by the pure slurry soaking method than for that obtained by the solid–liquid extraction method. Conversely, the pH value of porous ecological concrete with a microbial grouting film is reduced to approximately 8 at an age of 56 days. The compressive strengths of the porous ecological concrete specimens with the two films are almost the same. The results of this study provide the necessary theoretical basis for developing alkali reduction technology for porous ecological concrete with environmental and economic benefits.

## Introduction

Sodium silicate, polyurethane epoxy resin and cement are commonly used as cementing materials for the preparation of porous ecological concrete. Most of the above mentioned cementitious materials are toxic, and their safety hazards raise concerns. A large volume of ore is needed for their development, resulting in natural resource waste, environmental pollution and high energy consumption^1–3^. In addition, the cement hydration reaction releases alkaline substances, which increase the pH value of ecological concrete to approximately 12, and the levels of decrease in the amplitude and speed of the reaction are very small^4^. The research shows that for concrete cured for more than 30 days without alkali reduction measures, the pH value of a pore fluid can be maintained at approximately 11 for a long time. Moreover, vegetation cannot grow normally in such a high-alkalinity environment. When the pH values of planting concrete pores drop to approximately 11, a plant can grow^5^. The research of Gao et al. showed that after alkali reduction, the pH value of the pore fluid of low-alkaline cementing material remains stable at approximately 10 for a long time, and the strength of the material is reduced. When the solution of boric acid is no more than 5%, the pH value of foamed concrete is approximately 9. Wax sealing and carbonized coated ecological concrete has good effect of alkali reduction in the case of a tightly wrapped film and in most cases, carbonized coated ecological concrete the compressive strength of the concrete is basically unchanged. However, in practice, the wax sealing treatment is difficult to operate and the durability of paraffin remains to be studied, it is difficult to apply in practical engineering and carbonized treatment needs to be implemented with an advanced management technique. When the batches of carbonization coatings on the planting concrete specimens can make this combined method applicable to large-scale projects. Once the coating is damaged and unrecyclable, it cannot reduce alkali, the cost of the batch carbonization coating device is increased, and the repair coating process is more complicated^6–8^. Therefore, seeking new building materials and devices that can reduce the pore alkalinity of porous ecological concrete in an environmentally friendly and economical manner has become an urgent problem that needs to be solved in engineering.

Microbial calcium carbonate precipitation is a favourable approach due to its environmental protection and economic benefits. The principle of this technique is that some products of the microbial metabolism and some ions or mixtures of the external environment have physical and chemical reactions, resulting in the subsequent mineral deposition of metabolic by-products^9^. The microbial mineralization process with carbonate as a product is called microbially induced carbonate precipitation (MICP)^10^. Bacillus pasteurii, which is commonly used, is a nonpathogenic bacterium with high biological activity under acidic and alkaline conditions, high-salinity conditions and other harsh environmental conditions. This bacterium can use its own metabolism to generate urease, which can first hydrolyse urea and then produce ammonia root ions by dissolving ammonia in water to improve the pH value of the environment, causing Ca^2+^ and ${{\text{CO}}}_{3}^{2-}$ to react to form calcium carbonate precipitates^11^. Based on its advantages, MICP has been reported in the fields of foundation reinforcement, slope stabilization, soil liquefaction prevention, building material and cultural relic restoration, desert prevention and control, and water purification^12^. However, there are few reports on microbial grouting films and alkali-reducing porous ecological concrete. In addition, the vertical grouting method using microbial precipitation technology can easily cause uneven consolidation of the sand body, blockage of the grouting port, and reduction in the compressive strength. However, after grouting of the specimen is completed, the holes left inside by the introduction of the grouting pipe lead to a decrease in the compressive strength. The microbial transverse grouting method can eliminate the above problems, such as uneven consolidation, clogging of the grouting port, and voids in the specimen. After grouting is completed, the bacterial liquid is directly discarded, failing to be recycled and creating pollution and waste. The process of microbial cementation cannot be completed in the same integrated device, and the exposure of the test sample to air, the performance of multistep operations, and the replacement of the device may cause microbial species variation due to the introduction of miscellaneous bacteria, making it difficult to control the quality of the microbial cement. To solve this problem, we design and invent a device featuring a combined microbial film and a transverse grouting film. Additionally, there are few reports on the alkali reduction of porous ecological concrete coated by MICP technology. In this paper, microbial calcium carbonate precipitation technology is used to fabricate porous ecological concrete specimens by the microbial spraying method and the U-shaped/transverse grouting method (referred to as the microbial grouting film method). The pH values of porous ecological concrete specimens produced by the microbial grouting method and coat spraying method are tested by the solid–liquid extraction method and pure slurry soaking method. Then, the compressive strength is tested, providing an experimental and theoretical basis for the alkali reduction of film-coated porous ecological concrete using MICP technology. There are few reports on the above issues. In view of this research gap, we have designed and invented a coating device featuring a microbial film and a transverse/U-shaped grouting film that applies MICP technology for coating porous ecological concrete. We have applied to the China Intellectual Property Office for an invention patent. This device can be used to reduce the pore alkalinity of porous ecological concrete by the microbial grouting method and the spraying method.

## Microbial transverse/U-shaped grouting film device

### The principle of porous ecological concrete by microbial transverse/U-shaped grouting film device

We have designed and built a device and applied for an invention patent to the China Intellectual Property Office. This film-covered device provides a circulating apparatus and can be applied to test porous ecological concrete coated with a U-shaped, transverse, microbial grouting method.

The test device can integrate the reaction of microorganisms and mixed liquid, avoid the cumbersome operation process of moving the materials between different devices many times and prevent variations in the microbial liquid attached to the sample. The discharged microbial liquid and mixed liquid can be recycled, making the device environmentally friendly and economical. U-shaped and transverse grouting processes are realized from the outside to the inside to prevent the blocking and uneven reaction of the porous inner container. The porous ecological concrete manufactured by a membrane-covered device via the microbial grouting method provides a new concept for reducing the pore alkalinity of porous ecological concrete.

### Introduction microbial transverse/U-shaped grouting film device

**Figure 1:**
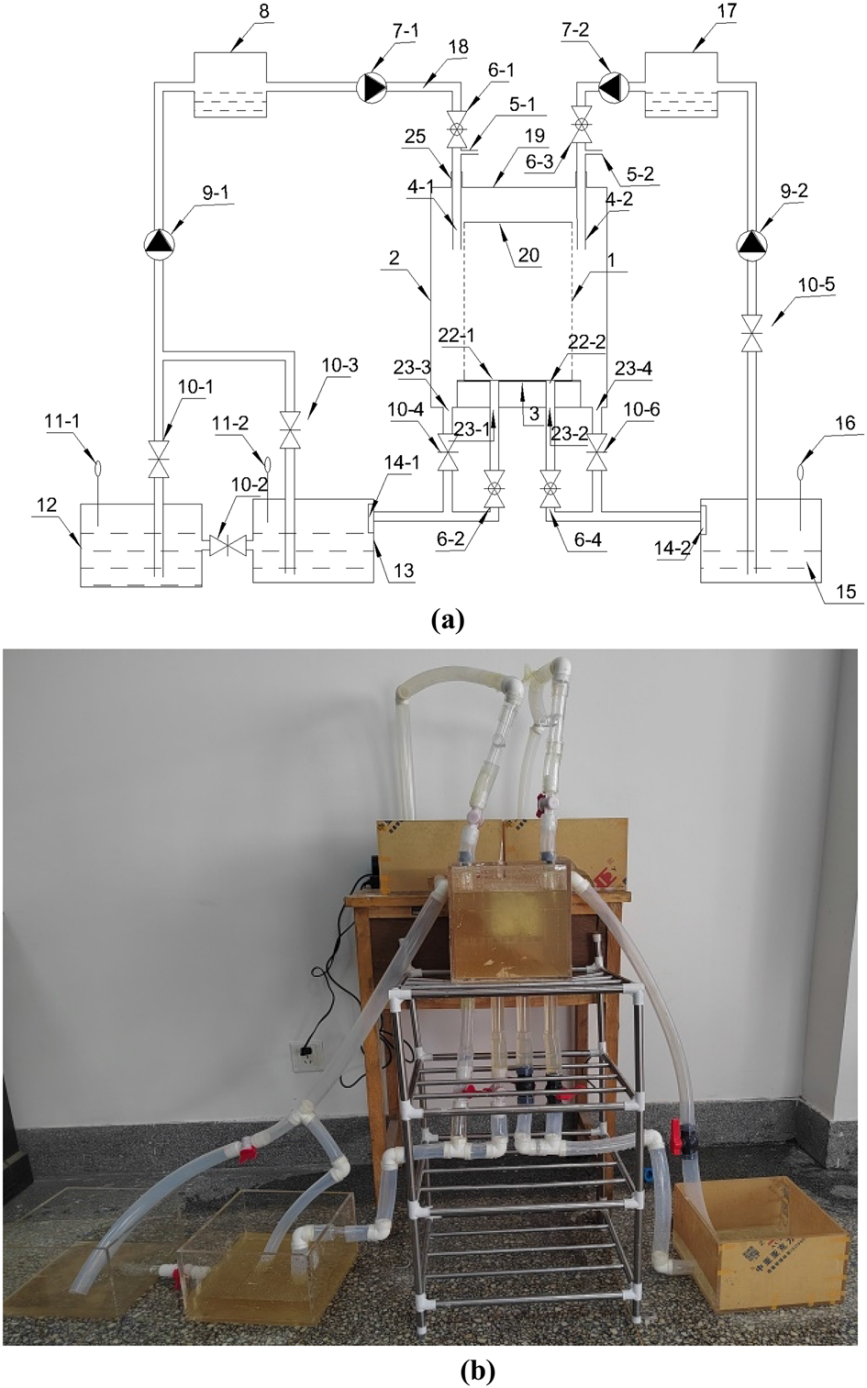
The drawing of microbial transverse/U-shaped grouting film device to porous ecological concrete: (**a**) schematic diagram; (**b**) experimental drawings. 1—Inner container, 2—outer container, 3—tray, 4-1—2 grouting pipe, 5-1—2 vent pipe, 6-1—4 flow valve, 7-1—2 suction pump, 8—microbial liquid storage tank, 9-1—2 circulating pump, 10-1—6 switch valve, 11–1—2 microbial concentration meter, 12—microbial incubator, 13—microbial liquid tank, 14-1—2 filter screen, 15—discharge mixed liquid tank, 16—mixed liquid concentration meter, 17—mixed liquid storage tank, 18—hose, 19—outer container top cover, 20—inner container top cover, 22-1—2 inner container bottom hole, 23-1—4 outer container bottom hole, 25—sealing pipe joint.

**Figure 2:**
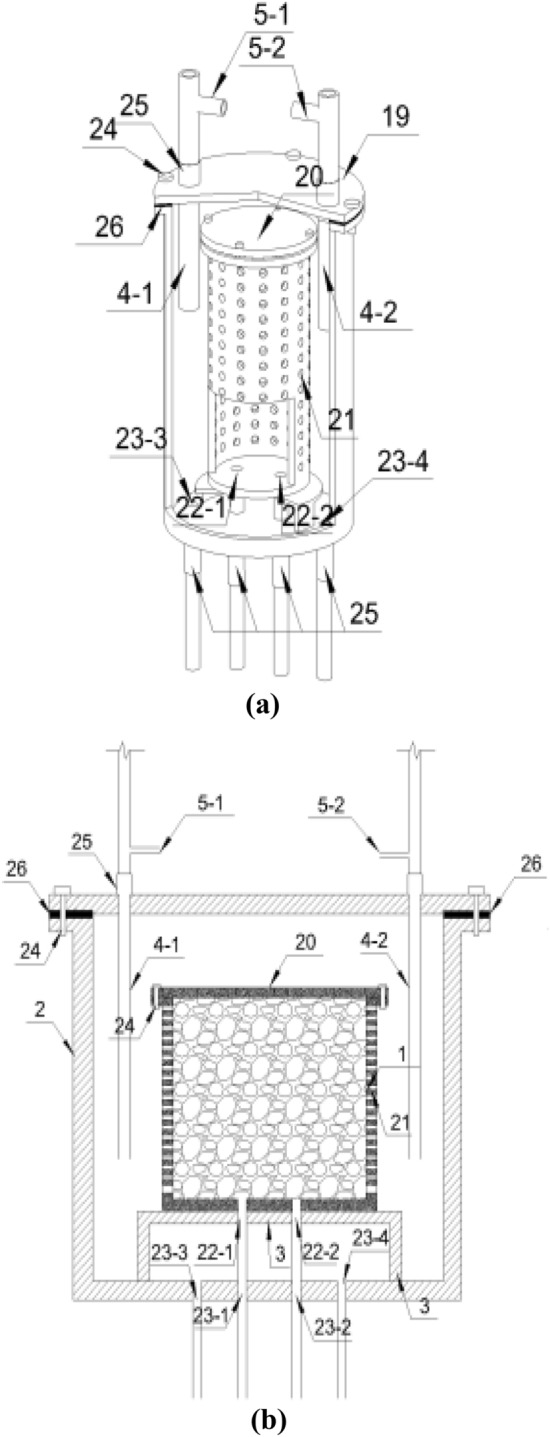
Schematic diagrams of the microorganism and mixed liquid reaction device: (**a**) axial section; (**b**) longitudinal section 1—Inner container, 2—outer container, 3 tray, 4-1—2 grouting pipe, 5-1—2 vent pipe, 19—outer container top cover, 20—inner container top cover, 21—Inner container side wall hole, 22-1—2 inner container bottom hole, 23-1—4 outer container bottom hole, 24—inner and outer container bolt hole and bolt, 25—sealing pipe joint, 26—inner and outer container gasket.

To reduce the pore alkalinity of porous ecological concrete, based on previous studies, we designed and invented microbial transverse/U-shaped grouting film device. The device structure mainly includes a microbial reaction–injection–discharge–circulation system and a mixed liquid reaction–injection–discharge–circulation system; the specific devices are shown in Figs. 1 and 2, respectively.

The microbial injection–reaction–discharge–circulation system is composed of a microbial injection-reaction system and a discharge–circulation system. The mixed liquid injection–reaction–discharge–circulation system is composed of a mixed liquid injection–reaction system and a discharge–circulation system. The microbial injection–reaction system is composed of hose 18, microbial liquid storage tank 8, suction pump 7-1, flow valve 6-1, vent pipe 5-1, grouting pipe 4-1, inner container 1 and outer container 2. The discharge circulation system is successively connected by hose 18, flow valve 6-2, switching valve 10-1—4, filter screen 14-1, microbial solution tank 13, microbial concentration measuring instrument 11-1—2, microbial incubator 12 and circulating pump 9-1; it can also be connected by microbial solution tank 13, microbial concentration measuring instrument 11-2, switching valve 10-3 and circulating pump 9-1. The mixed liquid injection reaction system is formed by successively connecting the mixture storage tank 17, hose 18, suction pump 7-2, flow valve 6-3, vent pipe 5-2, grouting pipe 4-2, inner container 1 and outer container 2. The mixed liquid discharge and circulation system is successively connected by discharge pipe hose 18, flow valve 6-4, switching valve 10-6, discharge mixed liquid pool 15, filter screen 14-2, mixed liquid concentration metre 16, switching valve 10-5 and circulating pump 9-2.

## Application of a microbial grouting film-covered porous ecological concrete specimen

### Preparation of porous ecological concrete

#### Raw materials for t﻿he preparation of porous ecological concrete

The cement is PO42.5 produced by Gansu Qilianshan cement plant Grade 5 cement, and various performance indices are shown in Table 1. The coarse aggregate is crushed stone with a single particle size of 16.5–31 mm produced by Gansu sand farm and the indices are shown in Table 2.

**Table 1:** Various performance indexes of coarse aggregate.

Particle size/mm	Apparent density (kg/m^3^)	Bulk density (kg/m^3^)
19.2–26.4	2678	1396

**Table 2:** Mix proportion of porous ecological concrete.

	Water cement ratio	Cement/(kg/m^3^)	Coarse aggregate/(kg/m^3^)	Water/(kg/m^3^)
Original sample	0.35	205	1640	72

#### Preparation of porous ecological concrete samples

After dry cleaning, the coarse aggregate concrete mixer is joined and stirred in 50% of the premixed water after waiting for wet stone cement to form. When the stone is wet, cement is added and stirred for 30 s. All the cement and the remaining water are added and stirred for 3 min, and then the mixture is poured into the mould three times. Each layer is compacted by vibration. The tamping rod is inserted spirally from the periphery to the center no less than 15 times to reduce the distance between particles. The dimensions of the test samples are 100 mm × 100 mm × 100 mm and 150 mm × 150 mm × 150 mm cubes. The test samples are demoulded after 24 h of pouring and forming, and they are set and cured for 3 days, 7 days, 14 days, 28 days and 56 days in a standard curing room. Then, the specimens are dried in a drying oven. The resulting samples are recorded as the originals, denoted by “Y” (“The original samples” is abbreviated as “Y” in the subsequent paper).

### Porous ecological concrete with microbial grouting films

#### Preparation of microorganisms

**Figure 3:**
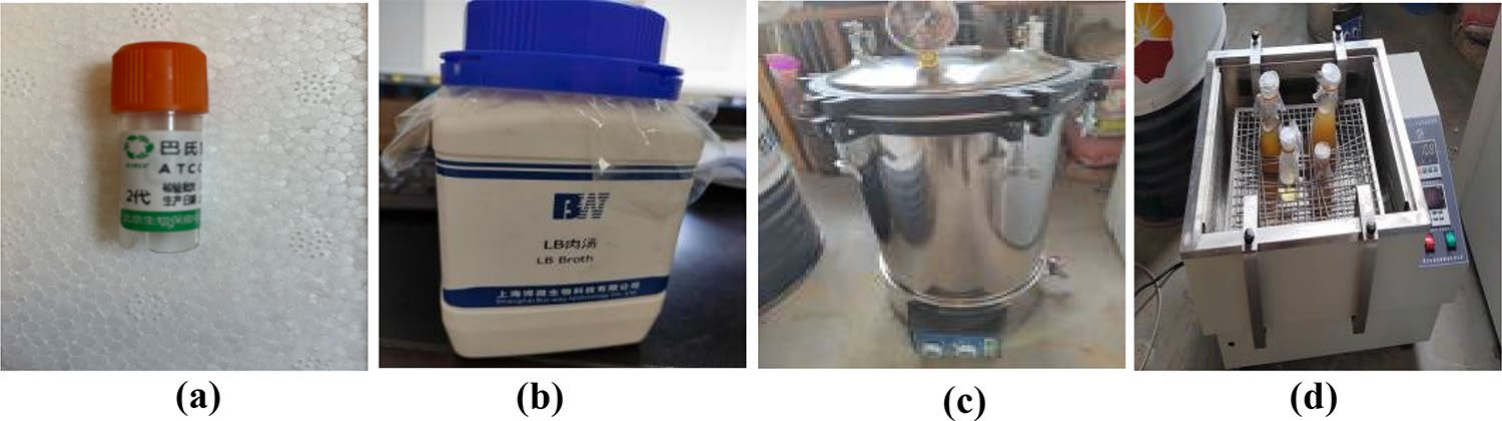
Expanded culture of Sporosarcina pasteurii: (**a**) Sporosarcina pasteurii; (**b**) LB broth; (**c**) Autoclave; (**d**) Constant temperature vibration incubator.

The preparation of microorganisms referred to in this paper mainly involves expanded cultures of bacteria, i.e., the expansion of glycerine-enclosed bacteria in lysogeny broth (Lysogeny brother is abbreviated as “LB broth” in the subsequent paper) and urea^13^. The bacterium selected in this paper is Sporosarcina pasteurii (ATCC 11859), which is purchased from the China Shanghai Conservation Biotechnology Centre (SHBCC) in Fig. 3a. LB broth and urea are used in the experiment. LB broth is from China Shanghai Bioway Technology Co., Ltd., and the formulation of the LB broth medium (1L) is shown in Table 3. The detailed preparation processes of Sporosarcina pasteurii and culture medium are described the section of Sample Preparation in Ref.^13^. To ensure that the experiment is carried out in a sterile environment, the above operations should be carried out on a sterile operation platform or under the action of alcohol disinfection as much as possible. The above operation process is shown in Fig. 3a–d.

**Table 3:** Ingredients of LB broth.

LB broth	Tryptone	Yeast powder	Sodium chloride
1L	10 g	5 g	10 g

#### Mixed solution preparation

The preparation of mixed solution in this paper mainly refers to the configuration of a nutrient solution composed of urea, calcium chloride and LB broth (3G/L). This solution provides urea and calcium ions for the MICP process and sufficient nutrition for the Sporosarcina pasteurii growth and reproduction processes. The ingredients of LB broth are shown in Table 3. A total of 60 g of urea (CH_4_N_2_O) and 110 g of anhydrous calcium chloride (CaCl_2_) are placed in 1 L of distilled water to prepare 1 mol/L mixed nutrient solution. Then, 3 g of LB broth is added to 1 L of mixed nutrient solution. The mixture needed for this experiment is obtained^13^.

#### Preparation of porous ecological concrete with a microbial grouting film

**Figure 4:**
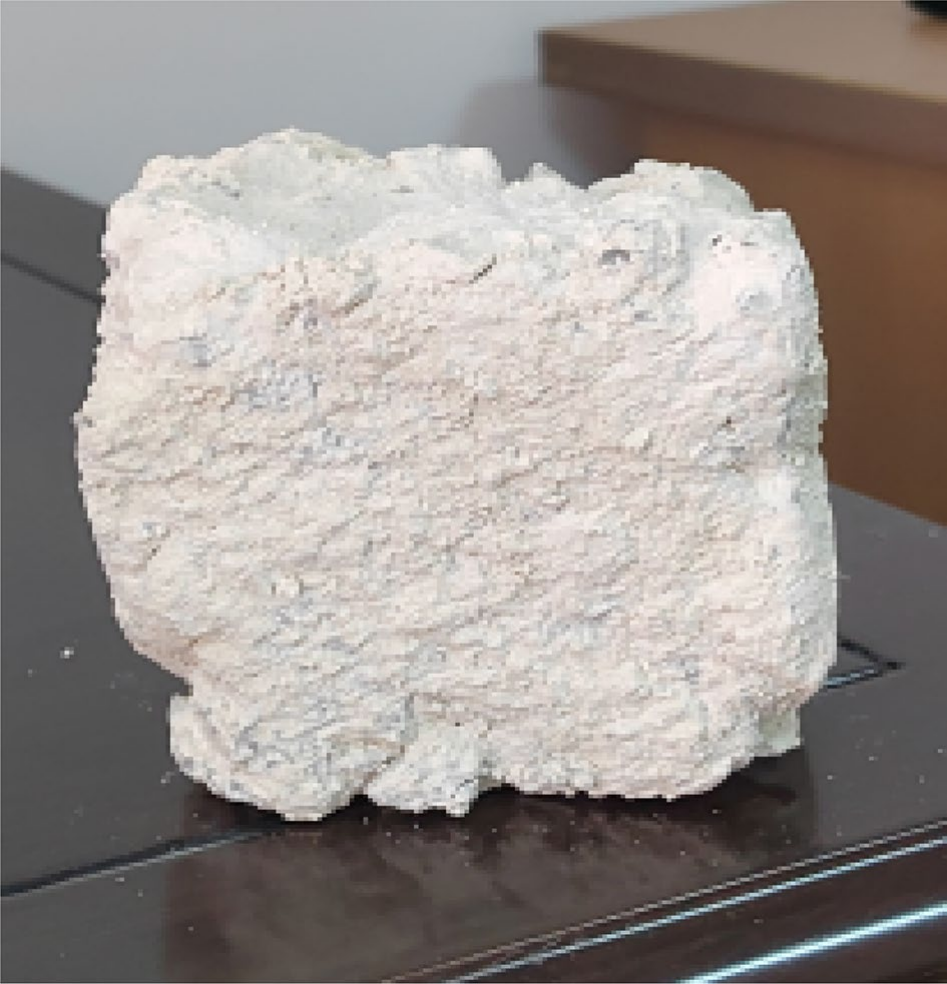
The diagram of GZ.

We adopt a spraying method and grouting method to prepare film-coated porous ecological concrete with reduced pore alkalinity. The homemade alkali reduction device for porous concrete coated with a microbial transverse grouting film is shown in Figs. 1 and 2. The steps are as follows. (1) The prepared porous ecological concrete is cleaned and placed in an internal reactor. (2) The urea and CaCl_2_ mixed solution injection–reaction–discharge–circulation system and Sporosarcina pasteurii bacterial solution discharge system are closed, while the Sporosarcina pasteurii circulation system with a bacterial solution concentration of OD = 1.5 is opened. After the reactor is filled and held for half an hour, the discharge system of Sporosarcina pasteurii is opened until the Sporosarcina pasteurii liquid volumes in the inner and outer containers are completely discharged. (3) The Sporosarcina pasteurii liquid injection and exclusion system is closed and the injection reaction system of urea and CaCl_2_ mixed solution is opened. The discharge circulation system of mixed solution is closed until urea and CaCl_2_ mixed solution fill the entire reactor, and it is opened after standing for half an hour. The urea and CaCl_2_ mixed solution discharge system can completely discharge the microbial liquid in the inner and outer containers. (4) Steps (1)–(3) are repeated 15 times successively until the test sample forms a protective layer of approximately 1 mm, the microbial bacterial solution and mixed solution are stopped to inject into the reactor, and porous ecological concrete with a microbial grouting film is dried naturally, and it is recorded as “GZ” (“Porous ecological concrete with a microbial grouting film” is abbreviated as “GZ” in the subsequent paper). The original porous ecological concrete specimens with ages of 3 days, 7 days, 14 days, 28 days and 56 days are grouted and covered with films, and the diagram of GZ are shown in Fig. 4.

### Preparation of porous ecological concrete with a microbial sprayed film

**Figure 5:**
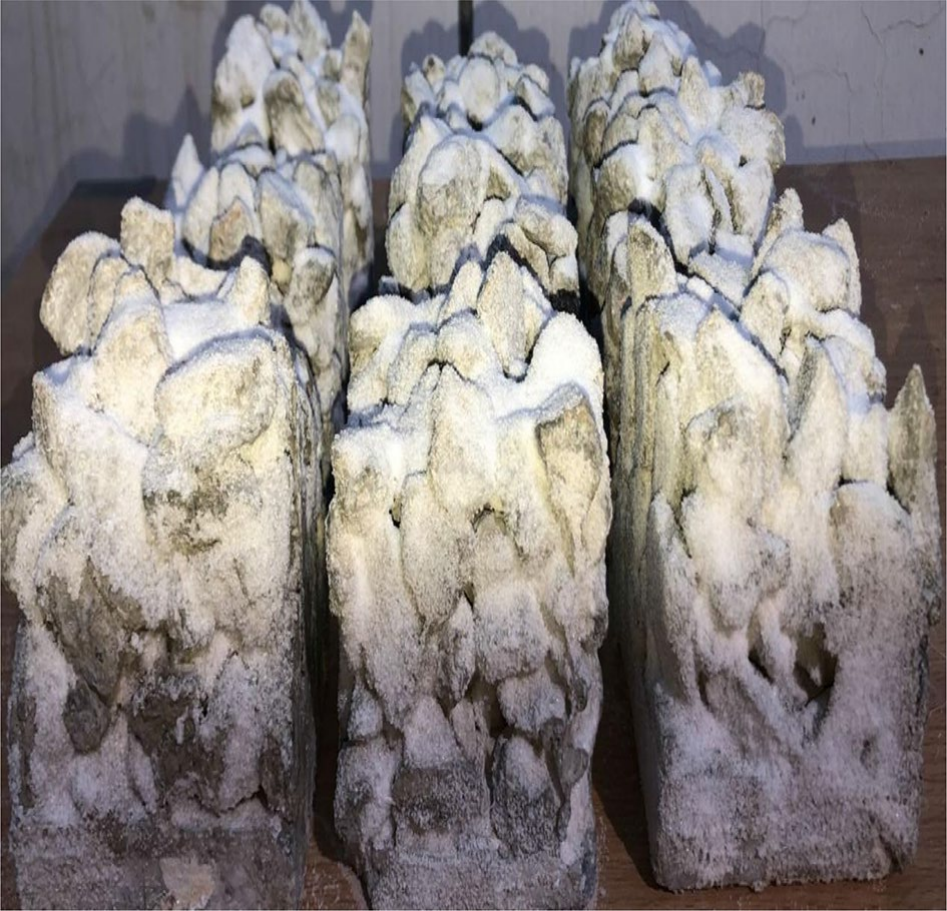
The diagram of PS.

The Sporosarcina pasteurii bacterial solution is prepared at an OD of 1.5 according to Section of “The principle of porous ecological concrete by microbial transverse/U-shaped grouting film device”, and the mixed solution prepared in Section of is evenly sprayed on each surface of the cube test block until the surface is saturated^13^. The samples are left to stand and sprayed every 6 h for a total of 9–15 times. The samples are naturally air-dried until there are no obvious droplets on the concrete surface, and a film of approximately 1 mm is formed, which is recorded as “PS” (“Porous ecological concrete with a microbial sprayed film” is abbreviated as “PS” in the subsequent paper). Porous ecological concrete specimens with ages of 3 days, 7 days, 14 days, 28 days and 56 days are sprayed and covered with films, and the diagram of PS is shown in Fig. 5.

### pH test and compressive strength test of porous ecological concrete

#### pH value test

Two groups of 150 mm × l50 mm × l50 mm specimens of porous ecological concrete with ages of 3 d, 7 d, 14 d, 28 d and 56 d are selected. The two groups of porous ecological concrete specimens with different ages are treated by either the grouting method or the spraying method. The solid–liquid extraction method and pure slurry soaking method are used^8^. The pH value of the filtrate is tested with a Shanghai Leici PHS-3C pH meter. The specific steps of the pH value test are as follows. (1)The pH value is measured by the solid–liquid extraction method. The porous ecological concrete test sample of 100 mm × 100 mm × 100 mm is ground, evenly mixed and sieved (0.08 mm square sieve), and 10 g of powder is weighed and placed in 100 g of distilled water to prevent carbonization with a rubber stopper and close the mouth of the test tube. For uniformity, the samples are shaken every 5 min and filtered with filter paper after 2 h, and their pH values are measured. (2) The sample pH value is measured by the pure slurry soaking method. The test sample with dimensions of 20 mm × 20 mm × 20 mm is immersed in a wide-mouthed bottle containing 2500 mL of distilled water. Then, the sample is sealed and soaked, and the pH value of the aqueous solution after soaking is tested, reflecting the alkalinity between pores.

#### Test of compressive strength

Three groups of porous ecological concrete specimens with microbial sprayed and microbial grouting films and dimensions of 150 mm × 150 mm × 150 mm are prepared after curing for 3 days, 7 days, 14 days, 28 days and 56 days to test their compressive strengths. Because porous concrete is formed by bonding between coarse aggregates, each surface of the test sample is in a state of aggregate extrusion and embedding after it is formed, and the flatness is poor. If the compressive strength test is carried out directly, the test block is not uniformly compressed or damaged along the surface bulge, resulting in a large deviation of the test results. To ensure the flatness of the concrete surface in the test, the test sample needs to be plastered 2 days before the test sample is cured to a specified age. Plastering is carried out on the relatively flat and nonformed surface of porous concrete. During this process, it is necessary to ensure that the plastering surface is smooth and flat. Moreover, it is important to scrape around the extruded excess slurry to ensure that the test sample is under uniform pressure. When plastering is finished, the test sample is returned to continue curing to a specified age. The compressive strength is tested by a YE-200T testing machine. When the upper and lower pressure plates are kept parallel in the measurement process, the loading speed is 0.3 MPa/s, and three samples are tested in each group. The measurement steps are carried out in accordance with the relevant provisions of GB/T50081-2019 “Standards for test methods of concrete physical and mechanical properties”.

## Results and analysis

### pH value test and analysis

**Figure 6:**
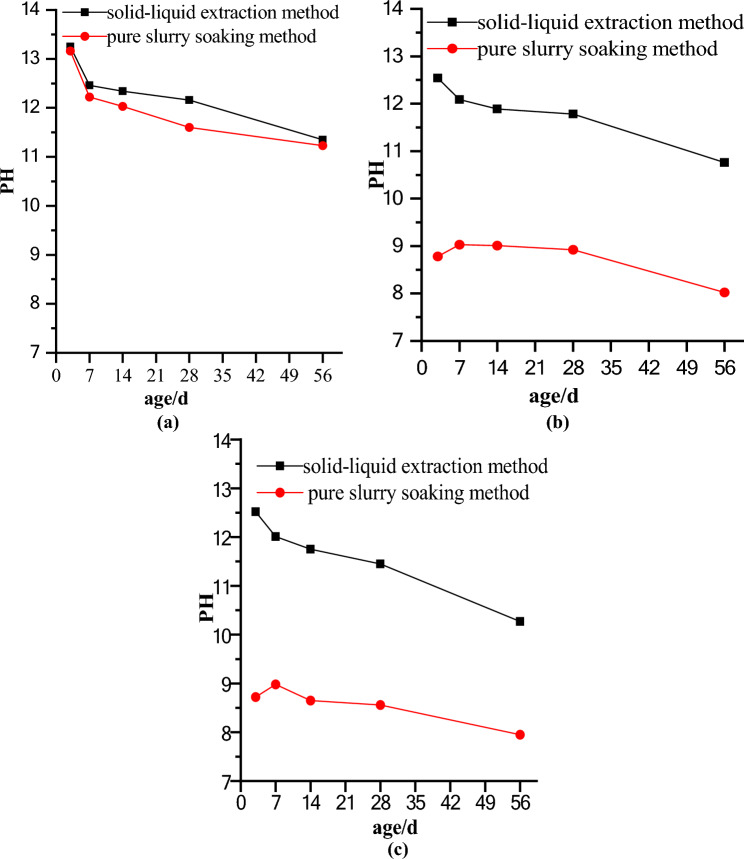
The pH values of the porous ecological concrete specimens covered by the same film type tested by the solid–liquid extraction method and pure slurry soaking method at different ages: (**a**) Y; (**b**) PS; (**c**) GZ.

The changes in the pH values with age for Y, GZ and PS tested by the solid–liquid extraction method and pure slurry soaking method, respectively, are shown in Fig. 6a–c.

The pH value changes of Y, GZ and PS at the ages of 3 days, 7 days, 14 days, 28 days and 56 days, which are tested by the solid–liquid extraction method and pure slurry soaking method, respectively, are shown in Fig. 6a–c. Figure 6a–c show that, on the one hand, the pH value measured by the solid–liquid extraction method is higher than that measured by the pure slurry soaking method for Y, GZ and PS at ages of 3, 7, 14, 28 and 56 days. On the other hand, the pH values of Y, GZ and PS decrease with increasing age in general. However, the pH values of GZ and PS tested by the pure slurry soaking method first increase slightly and then decrease with age.

The changes in the pH values of Y tested by the solid–liquid extraction method and pure slurry soaking method at ages of 3 days, 7 days, 14 days, 28 days and 56 days are shown in Fig. 6a. (1) From the pH test, the pH values obtained by the solid–liquid extraction method are significantly higher than those obtained by the pure slurry soaking method at the same age. (2) Y are tested by the solid–liquid extraction method and pure slurry soaking method, and the pH values of Y exceed 13 at 3 days and gradually decrease with increasing age. At 56 days, the pH value is still more than 11, which is strongly alkaline and not conducive to plant growth. On the one hand, many calcium hydroxides and precipitates on the surface of cement stone are generated in the process of cement hydration. On the other hand, the raw materials for cement production contain alkaline substances generated by the plasma hydrolysis of Na^+^ and K^+^. However, with the gradual slowing of the hydration reaction rate and the carbonation effect of carbon dioxide in air on concrete, the pH value gradually decreases^14^.

The pH values of the porous ecological concrete specimens covered by the same film type tested by the solid–liquid extraction method and pure slurry soaking method at ages of 3 days, 7 days, 14 days, 28 days and 56 days are shown in the change diagrams in Fig. 6b,c. Figure 6b shows the pH values of PS at different ages resulting from tests by the pure slurry soaking method and solid–liquid extraction method. The pH value resulting from the solid–liquid extraction method decreases with age. The change trend of the pH value is the same as that of Y (Fig. 6a). After 56 days, the minimum pH value is greater than 10; thus, the pH value measured by this method is not suitable for plant growth. With increasing age, the pH value obtained by the pure slurry soaking method first increases from 3 to 7 days and then decreases from 7 to 56 days, and the change trend is relatively flat. This change trend of pH needs to be further studied. The two different pH testing methods result in different change trends of the pH values of the pore solutions of porous ecological concrete specimens with the same films. This phenomenon is explained below, but it is necessary to study the microstructure of the microbial calcium carbonate precipitate.

The pH values of GZ determined by the pure slurry soaking and solid–liquid extraction methods are shown in Fig. 6c. (1) For GZ, the change trend of the pH value obtained by the solid–liquid extraction method is the same as the values of Y and PS, as shown in Fig. 6a,b. (2) The pH value obtained by the pure slurry soaking method first rises from 3 to 7 days and then decreases from 7 to 56 days. In addition, the change trend is relatively gentle. The reason for this phenomenon needs to be further studied. The pH value of porous ecological concrete with a microbial grouting film is approximately between 8 and 9, basically meeting the needs of plant growth. (3) Fig. 6b,c show that the pH values measured by the solid–liquid extraction method are significantly higher than those measured by the pure slurry soaking method for GZ and PS.

**Figure 7:**
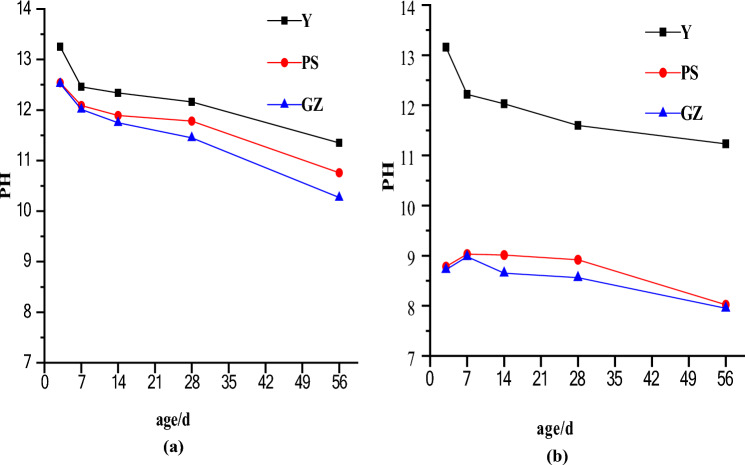
The pH values of Y, GZ and PS obtained by the same pH test method at different ages: (**a**) by solid–liquid extraction method, (**b**) by pure slurry soaking method.

The pH values of Y, GZ and PS obtained by the same pH test method at different ages are shown in Fig. 7a,b. Figure 7a shows that the pH values of Y, GZ and PS measured by the solid–liquid extraction method decrease at the same age; however, the decrease is not particularly obvious. At the same age, the pH value of GZ is slightly lower than that of PS, but the pH value is still above 10, which is not conducive to plant growth. Figure 7b shows that the pH values of Y at the same age, measured by the pure slurry soaking method, are quite different from those of GZ and PS. In addition, the pH value of GZ is slightly lower than that PS at the same age. After 56 days, the pH values of GZ and PS are approximately 8, basically satisfying the requirements of plant growth. The pH values obtained by the two test methods are quite different. The reason for this difference is that the solid–liquid extraction method destroys the protective film on the concrete surface when the concrete is ground into a powder. After the concrete is broken, it is dissolved 10 times in distilled water, artificially promoting the dissolution rate of alkaline hydrate and accelerating the action of the concrete in water. Therefore, the pH value measured by the solid–liquid extraction method is higher than that measured by the pure slurry soaking method, and it is alkaline. The pure slurry soaking method does not damage the test sample and retains its integrity. The microbial protective films generated by the spraying method and grouting method on the concrete surfaces are not damaged and can continue to isolate alkaline hydrates. Therefore, the obtained pH value is low from the pure slurry soaking method. After treating the microbial protective films formed by the spraying method and grouting method, the pH values of porous ecological concrete at 7–56 days tested by the pure slurry soaking method decrease significantly within the range of normal plant growth. In addition, when planting in porous ecological concrete, the pH value in the plant growth environment is the result of the long-term action of the concrete and the surrounding water environment. This action should be a slow leaching process. The intact test sample is soaked in distilled water by the pure slurry soaking method, the pH of the tested aqueous solution is relatively close to the actual value, and the pH value may be low. Stable protective films on the surfaces of porous ecological concrete specimens are formed by the grouting method and spraying method to seal alkaline hydrate in cement stone to effectively reduce the pH value of the planting environment. This result is consistent with the research of Wang et al., which concerned the effect of the silane soakage technique on the alkali reduction of macroporous ecological concrete^14^.

### Compressive strength of porous ecological concrete

**Figure 8:**
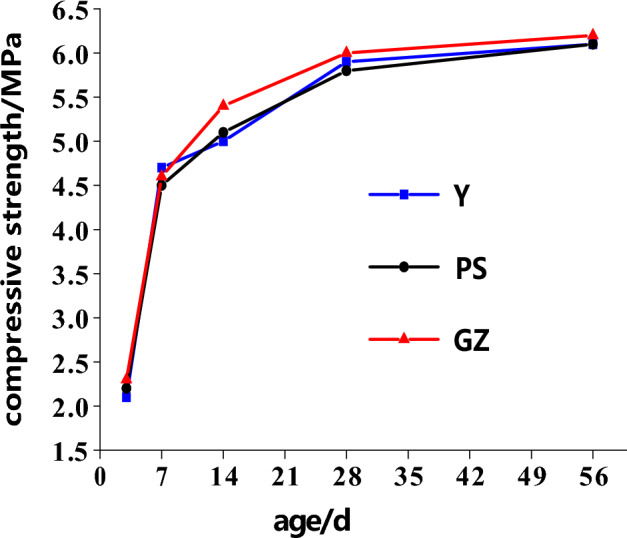
The compressive strength values of Y, GZ and PS change at different ages.

The compressive strength values of Y, GZ and PS change at different ages, as shown in Fig. 8. Figure 8 shows that the compressive strength values of Y, GZ and PS gradually increase with increasing age. The compressive strengths of GZ and PS are basically the same as those of Y at the same age. Due to the special skeleton structure, the strength determinants and variation trends of porous ecological concrete are not the same as those of ordinary concrete. The strength of porous ecological concrete is mainly determined by the mechanical biting force between aggregates, the strength of the cementation layer, the interfacial bonding strength between the cementation layer and the aggregate, and the number of bonding points, among other factors^15^. Only the porous ecological concrete specimens covered by microbial grouting and microbial sprayed films act on the surface of ecological concrete. These specimens do not affect the aggregate structure or cementation strength. Therefore, the strength differences among Y, GZ and PS are very small at the same age, indicating that the films are only attached to the surfaces of the porous ecological concrete specimens. Moreover, the films do not affect the overlapping or cementation of aggregate, and they do not change the strength.

## Conclusions

The pore solution of porous ecological concrete is highly alkaline, and plants cannot grow. Traditional porous concrete film materials may lead to resource waste, poor alkali reduction effects, high costs and complicated processes, which are difficult to achieve in engineering. In this paper, by using environmentally friendly and economical microbial calcium carbonate precipitation approaches, an integrated device with a microbial film and a transverse/U-shaped grouting film is designed and manufactured. The device is used to grout porous ecological concrete to reduce the pore pH value. The device overcomes the problems of blockage of the grouting port caused by the vertical grouting of microorganisms. In addition, the holes are left in the interior of the test piece when the grouting pipe is introduced. The compressive strength of the test piece is reduced, the utilization of microorganisms is optimized, and the variation is reduced. The reduction in pore alkalinity of the porous ecological concrete with a microbial grouting film resulting from this device is larger than that of the concrete with a spraying film. Furthermore, by using the pure slurry immersion method, the pH value of porous ecological concrete with a microbial grouting film is reduced to approximately 8 at 56 days, which is suitable for plant growth. The main research equipment and experimental results in this paper are as follows:

1. A combined microbial film and transverse/U-shaped grouting film device is designed and invented. We have applied for an invention patent to the China Intellectual Property Office. The main structure includes a microbial and a mixed liquid injection–reaction–discharge–circulation system. The device is used to cover the film for porous ecological concrete to reduce pore alkalinity.
2. Porous ecological concrete specimens with microbial grouting films and microbial sprayed films are prepared.
3. When the age is 3 days, 7 days, 14 days, 28 days or 56 days, the pH value measured by the solid–liquid extraction method is higher than that obtained by the pure slurry soaking method. The pH values of the original samples, porous ecological concrete with a microbial grouting film and porous ecological concrete with a microbial sprayed film basically decrease with increasing age. The pH values of porous ecological concrete specimens with microbial grouting films and microbial sprayed films first increase and then decrease with increasing age. The pH value of porous ecological concrete with a microbial grouting film is slightly lower than that with a microbial sprayed film. Conversely, the pH values of porous ecological concrete specimens with microbial sprayed films and microbial grouting films are still more than 10. This value is not conducive to plant growth. The pH value of the original sample at the same age measured by the pure slurry soaking method is quite different from those of porous ecological concrete specimens with microbial grouting films and microbial sprayed films. In addition, the pH value of porous ecological concrete with a microbial grouting film is slightly lower than that with a microbial sprayed film at the same age. The pH values of the porous ecological concrete specimens with microbial grouting films and microbial sprayed films are approximately 8, which can basically meet the plant growth requirements. The pH results obtained by the two different pH test methods are quite different.
4. The results show that the compressive strengths of porous ecological concrete specimens with microbial grouting films and with microbial sprayed films are basically the same as that of the original sample at the same age. Therefore, the compressive strengths of porous ecological concrete specimens with microbial grouting films and with microbial sprayed films have not been improved.

We provide a theoretical and experimental basis for porous ecological concrete with microbial alkali reduction measures. Of course, there is still a certain gap between the research results and practical applications. In the future, on the basis of the research on microbial porous concrete film, the engineering culture and the reproduction of the bacteria needed for MICP film coating can be carried out. Moreover, we can conduct a microscopic study on the MICP film coating process, and the MICP film coating process can be improved to be in agreement with the actual construction requirements.

## Data Availability

The datasets used and/or analysed during the current study available from the corresponding author on reasonable request.
